# Predominant Yeasts During Artisanal Mezcal Fermentation and Their Capacity to Ferment Maguey Juice

**DOI:** 10.3389/fmicb.2018.02900

**Published:** 2018-12-06

**Authors:** Hipócrates Nolasco-Cancino, Jorge A. Santiago-Urbina, Carmen Wacher, Francisco Ruíz-Terán

**Affiliations:** ^1^Departamento de Alimentos y Biotecnología, Facultad de Química, Universidad Nacional Autónoma de México, Ciudad Universitaria, Ciudad de México, Mexico; ^2^Facultad de Ciencias Químicas, Universidad Autónoma Benito Juárez de Oaxaca, Oaxaca, Mexico; ^3^División de Dirección de Carrera de Agricultura Sustentable y Protegida, Universidad Tecnológica de los Valles Centrales de Oaxaca, Oaxaca, Mexico

**Keywords:** Mezcal, non-*Saccharomyces*, *Saccharomyces cerevisiae*, maguey juice fermentation, volatile compounds

## Abstract

Artisanal mezcal is produced by the natural fermentation of maguey juice, which frequently results in a process that becomes stuck or is sluggish. Using selected indigenous starter inoculums of *Saccharomyces* and non-*Saccharomyces* yeasts is considered beneficial in overcoming these problems and thereby preserving the essence of the artisanal process. In this work, three hundred and four yeast isolates were recovered from 17 distilleries and then grouped by the ARDRA analysis, their restriction profiles were clustered in 15 groups. Four of them included 90% of all isolates, and these were identified using the sequence of the D1/D2 domain of the large-subunit rDNA. *Pichia kudriavzevii, Pichia manshurica, Saccharomyces cerevisiae*, and *Kluyveromyces marxianus* were detected as predominant species. Both species belonging to the *Pichia* genus were detected in 88% of the distilleries, followed by *S. cerevisiae* (70%) and *K. marxianus* (50%). In order to evaluate the fermentative capacity, one strain of each species was assessed in a pure and mixed culture in two culture media, filtered maguey juice (MJ) and maguey juice including its bagasse (MJB). Findings demonstrated that non-*Saccharomyces* yeast presented better growth than that of *S. cerevisiae*. *K*. *marxianus* PA16 was more efficient for ethanol production than *S. cerevisiae* DI14. It produced 32 g/L of ethanol with a yield of 0.47 g/g and efficient of 90%. While, *P. kudriavzevii* produced more ethyl acetate (280 mg/L) than the others species. All fermentations were characterized by the presence of isobutyl and isoamyl alcohol. The presence of *K. marxianus* in a mixed culture, improved the ethanol production and volatile compounds increased using co-cultures.

## Introduction

Mezcal is a Mexican alcoholic beverage distilled from the fermented juice of cooked mature maguey plants (stem). It is produced from several species of maguey in the territory protected by the Appellation of Origin Mezcal [AOM; NOM-070-SCFI-2016, Norma Oficial Mexicana: Bebidas Alcohólicas-Mezcal-Especificaciones (NOM-070-SCFI-2016, 2017)]. In recent years this typical Mexican beverage has garnered increased attention in international markets and improved in quality (Mezcal Regulatory Council, 2018). In 2017, 3 million liters were exported to 60 countries of 4 million mezcal liters commercialized under the AOM. This spirit is produced, bottled and marketed according to Mexican regulations (NOM-070-SCFI-2016, 2017). Mezcal is classified under three categories known as Ancestral Mezcal, Artisanal Mezcal, and Mezcal, all three categories must meet the following four stages in the elaboration process: cooking maguey, milling cooked maguey, fermentation, and distilling. During cooking, maguey fructans are hydrolyzed into simple sugars; this process is performed in pit oven over 3 to 5 days. In ancestral and artisanal mezcal production, pulp, and fibers (bagasse) obtained by the milling of cooked maguey are placed in wooden vats and left to sit over 2 or 3 days, warm water (40°C) is then added. Here, the maguey juice undergoes natural fermentation, which relies on the microbiota present in the production environment, including bacteria and yeasts (Lachance, 1995; Narváez-Zapata et al., 2010; Verdugo Valdez et al., 2011; Páez-Lerma et al., 2013; Kirchmayr et al., 2017). The crushing equipment and the wooden vats have been reported as the main source of inoculum (Lachance, 1995). Some studies have found that *Saccharomyces cerevisiae, Kluyveromyces marxianus*, and *Torulaspora delbrueckii* are common yeast species in Mezcal and Tequila fermentations (Lachance, 1995; Verdugo Valdez et al., 2011; Páez-Lerma et al., 2013), whereas *Pichia kluyvery, Zygosaccharomyces bailii, Clavispora lusitaniae, Candida ethanolica, Saccharomyces exiguous, Candida diversa, Pichia fermentans*, and *Hanseniaspora guilliermondii*, among others, are present depending on the geographical area of production (Verdugo Valdez et al., 2011; Páez-Lerma et al., 2013). Until now, yeast populations involved in mezcal fermentation in most states and regions with AOM remains unknown. Knowledge of indigenous yeast population is an important step in the exploration of yeast species and can be used as starters in alcoholic beverage production, contributing to the preservation of the regional typical characteristics and the improvement of the organoleptic quality of the final product (Sun et al., 2014; González-Robles et al., 2015).

The natural fermentation of mezcal has resulted in a slow or arrested process, which can vary from 8 to 30 days. This fermentation process is characterized by the low ethanol yields and a fermentation efficiency of around 30 to 40%, as well as varying concentrations of residual sugars (10–60 g/L; Nuñez-Guerrero et al., 2016; Kirchmayr et al., 2017; Mezcal Regulatory Council, 2018). To overcome these problems, some starter cultures have been designed and proposed, using pure or mixed cultures of *S. cerevisiae* and non-*Saccharomyces* yeasts (*Kloeckera apiculata, Kloeckera vinae, K. marxianus, T. delbrueckii, P. kluyvery*) isolated from fermented maguey juice (Díaz-Montaño et al., 2008; González-Robles et al., 2015; Segura-García et al., 2015; Nuñez-Guerrero et al., 2016). Mezcal non-*Saccharomyces* yeast such as *K. marxianus* and *P. kluyveri* are characterized by their ability to produce esters (Segura-García et al., 2015). Thus, different studies have concluded that mixed cultures provide a positive impact on sensorial characteristics of the mezcal, thanks to a greater richness of volatile compounds and the good yield produced (González-Robles et al., 2015; Nuñez-Guerrero et al., 2016). Despite these studies, the influence of different yeast species in pure and mixed cultures on the flavor production are not clear, and the impact of bagasse on the volatile compounds and ethanol production has not been investigated.

The Official Mexican Regulation establishes that mezcal fermentation can be performed naturally or by using inoculated microorganisms (NOM-070-SCFI-2016, 2017). In order to take advantage of this, information is required to understand natural fermentation and to select starter cultures. Most distilleries are located in rural areas and they normally do not use starter cultures, due to the lack of information, availability and providers of inoculum. The aim of the present work was to identify the predominant yeast species associated with the natural fermentation of maguey juice for mezcal production in Oaxaca State (the most important mezcal-producing state and leading exporter within the territory protected by the Appellation of Origin of Mezcal; Mezcal Regulatory Council, 2018), including regions that have never been studied before, to further evaluate the fermentative capacity and the formation of volatile compounds in maguey juice, influenced by fermentation temperature, inoculum (pure and mixed), and bagasse. This study will contribute to the knowledge and understanding of their role in maguey fermentations. It is also important to promote the appreciation of this Mexican beverage as a cultural heritage.

## Materials and Methods

### Study Site

**Figure 1:**
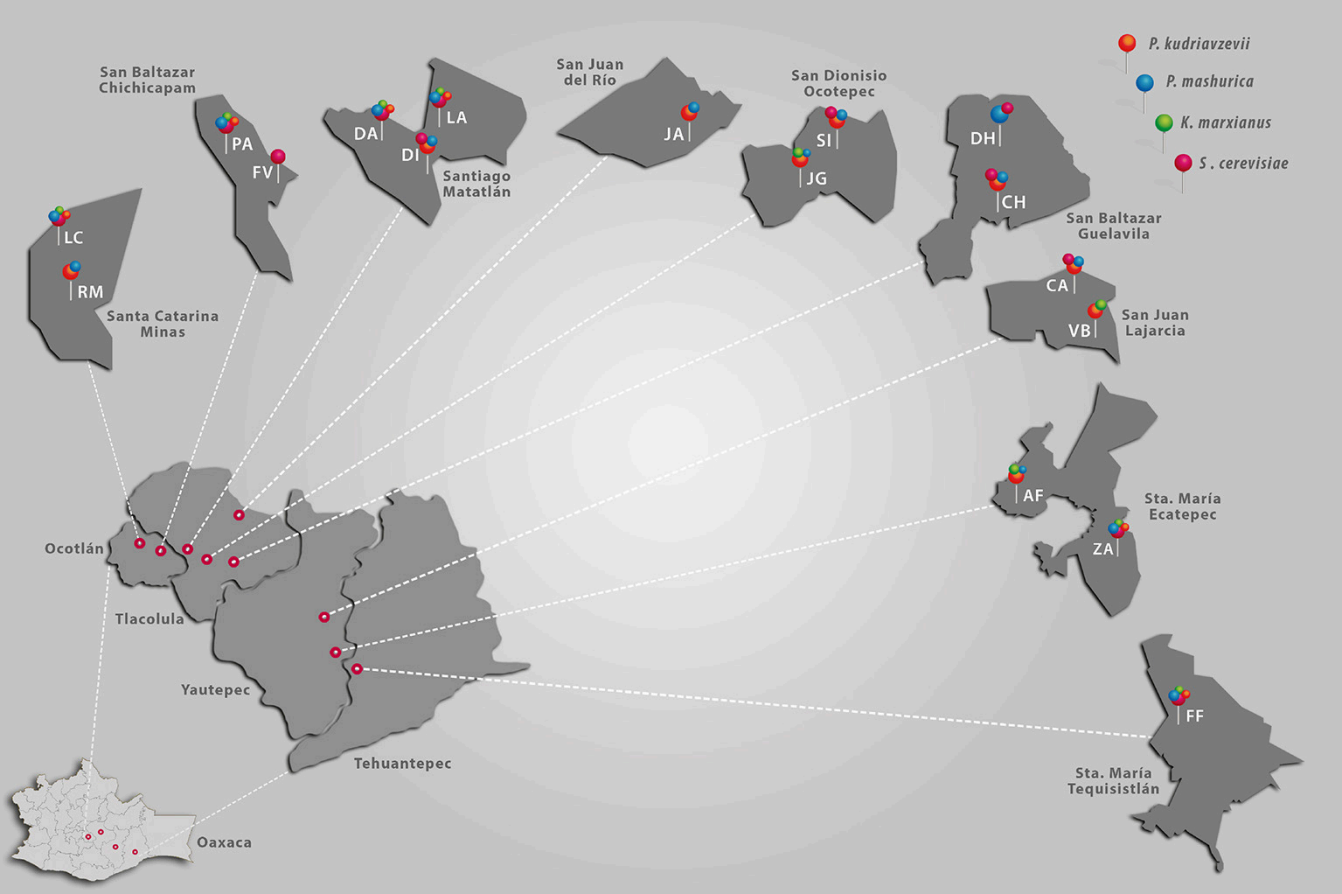
A map depicting the locations of the distilleries sampled and the occurrence of the four predominant yeast species. Ocotlán, Yautepec, Tlacolula, and Tehuantepec are districts of Oaxaca State. Santa Catarina Minas, San Baltazar Chichicapam, Santiago Matatlán, San Juan del Rio, San Dionisio Ocotepec, San Baltazar Guelavila, San Juan Lajarcia, Santa (Sta.) María Ecatepec, and Santa María Tequisistlán are municipalities in each district. The letters RM, LC, PA, FV, DA, DI, LA, JA, SI, JG, DH, CH, CA, VB, AF, ZA, and FF correspond to the symbology assigned to each distillery.

The samples were collected from 17 distilleries distributed across 9 communities located in the Ocotlán, Tlacolula, Tehuantepec, and Yautepec districts of Oaxaca State, Mexico (Figure 1). The distance between distilleries ranged between 10 m and 240 km.

### Fermented Maguey Juice Sampling and Yeast Isolation

Samples of fermented maguey juice, for the isolation of yeast were taken during the beginning, middle, and final stage of natural fermentation. One sample of a 100 mL was taken directly from the vats and spread-plated in duplicate, on plates of potato dextrose agar PDA (DIBICO, Mexico City, Mexico) adjusted to a pH 3.5, using tartaric acid 10% (w/v) solution. Immediately after returning to the laboratory, the plates were incubated at 30°C for 3–5 days. After the incubation period, at each stage of fermentation (initial, middle, and final) three colonies per morphology were selected for sub-culturing in YEPD agar (10 g/L yeast extract, 20 g/L peptone, 20 g/L dextrose, 15 g/L agar). The purified isolates were suspended in YEPD broth containing 30% (v/v) glycerol and stored at −20°C until identification.

### Amplified Ribosomal DNA Restriction Analysis (ARDRA) of Isolated Cultures and Grouping

Yeast DNA was extracted as described by Santiago-Urbina et al. (2015). The D1/D2 divergent domain of the large subunit (26S) rRNA gene of the isolated yeast, was amplified by PCR with NL1 and NL4 primers and the conditions described by Kurtzman and Robnett (1998). PCR products were digested with the restriction enzymes *Hae* III, *Hinf* I, and *Hha* I (Invitrogen, CA, USA) and subsequent visualized steps were performed as described by Santiago-Urbina et al. (2015). Band sizes were calculated with reference to a 100 bp ladder (DNA Ladder, Invitrogen). The size of fragments smaller than this standard DNA marker were not estimated. Isolates were grouped based on their ARDRA patterns using an unweighted pair group average (UPGMA) cluster analysis based on the Jaccard Similarity Index using the PAST software version 2.17c.

### Sequence Analysis of the 26S rDNA D1/D2 Domain

Partial sequencing of the (26S) rDNA was performed on representative strains of each group with the largest number of isolates. The NL1 and NL4 primers were used to amplify the D1/D2 domain following the conditions described in Kurtzman and Robnett (1998). The amplified fragments were then sequenced by Macrogen Inc. (Seoul, Korea). The sequences were compared with those available in the GenBank database at http://www.ncbi.nlm.nih.gov/nucleotide using the basic local alignment search tool (BLAST). The nucleotide sequences have been deposited in GenBank under accession numbers: KT875209, KT875210, KT875211, and KT875208.

### Fermentative Capacity

#### Yeast Strains

From the four predominant yeast species, two non-*Saccharomyces* (*P. kudriavzevii* JA10 and *K. marxianus* PA16) and *S. cerevisiae* (DI14) were used to determine their fermentative capacity on *Agave angustifolia*. *P. kudriavzevii* JA10 was isolated from the Alipus San Juan distillery located in San Juan del Río, Tlacolula, Oaxaca; *K. marxianus* PA16 was isolated from the mezcal pierde almas distillery located in San Baltazar Chichicapan, Ocotlán, Oaxaca; *S. cerevisiae* DI14 was isolated from the mezcal Don Isaac distillery located in Santiago Matatlán, Tlacolula, Oaxaca.

#### Fermentation and Culture Medium

Fermentation capacity was determined by cultivating pure and mixed strains in two culture media, filtered maguey juice (MJ) and maguey juice plus its bagasse (MJB). Concentrated juice (25°Brix) obtained from cooked maguey (*Agave angustifolia*) provided by the Real Matlatl distillery (Santiago Matatlán, Oaxaca, Mexico) was filtered, diluted with distilled water and adjusted at 10°Brix with a refractometer (Atago, Tokyo, Japan). The MJ medium consisted of 100 mL of filtered maguey juice at 10°Brix, and MJB medium was composed of 80 mL of filtered juice and supplemented with 30 g of residual bagasse obtained from maguey. Bagasse was washed with tap water to remove sugar residue (bagasse was added to mimic must conditions, such as is performed in ancestral and artisanal mezcal production process). Both culture media were sterilized at 121°C for 15 min. Fermentations were carried out in 125-mL Erlenmeyer flasks. The inoculation of the culture media was carried out to obtain an initial cell concentration of 1 × 10^7^ cells/mL. Mixed cultures were inoculated in a ratio of 1:1. Inoculum consisted of yeast grown in YEPD liquid medium overnight. Fermentations were performed at 30°C (this temperature was used to mimic the environmental conditions in distilleries) without agitation for 72 h and all experiments were carried out in duplicate. Every 12 h a sample was collected from each fermentation under sterile conditions and the main fermentation products, as well as the biomass were analyzed in triplicate. All metabolites (methanol, ethanol, higher alcohols, esters, and acetaldehyde) were determined at the end of the fermentation.

### Cell Counting and Carbohydrate Concentrations

Microscopic counts in the must were done using a Neubauer chamber. The reducing sugar concentration (fructose and glucose) was measured with a DNS (Dinitrosalicylic acid) reagent (Miller, 1959).

### Ethanol Determination

Ethanol content was determined as indicated by the Mexican norm (NMX-V-13-NORMEX-2013, 2014) as follows: 50 mL of the sample was diluted with 20 mL of water. It was then distilled and recovered in a 50 mL volumetric flask containing 4 mL of distilled water. After that, it was injected in a digital densitometer (Anton Paar, DMA 4100M, Switzerland).

### Headspace-Solid Phase Microextraction (HS-SPME)

Concentrations of higher alcohols (isobutyl, isoamyl and amyl alcohol, 1-propanol), esters (ethyl acetate, ethyl lactate), methanol, and acetaldehyde were determined using a head space solid phase microextraction (HS-SPME), followed by gas chromatography. First, 10 mL of the juice sample was mixed with 1 mL of 2-pentanol as an internal standard (20 mg/L). Then, to extract the volatile compounds, a poly dimethylsiloxane/Divinylbenzene (PDMS/DVB) fiber (Supelco, PA, USA) was used. A 1.7 mL sample was placed into a 5 mL headspace vial and 0.49 g of sodium chloride was added. After 5 min of equilibration at 25°C and agitation of 1,200 rpm, the fiber was inserted into the headspace and the solution was swirled in a magnetic stirrer at 12,000 rpm and 60°C for 30 min. The fiber was then transferred to the injector for desorption at 270°C for 15 min. Gas chromatography analysis was performed on a Shimadzu Plus 2010 GC system equipped with a flame ionization detector, using a DB-1301 column (60 m, 0.250 mm, film thickness, 1 μm, Agilent). The oven temperature was programmed at 40°C for 16 min, followed by a gradual increase of temperature at a rate of 5°C/min up to 60°C, and then raised to 260°C at a rate of 70°C/min. The gas carrier was hydrogen at a flow rate of 2.0 mL/min.

### Statistical Analysis

Significant differences among treatments cultures were determined for each volatile compound and ethanol yield, productivity, efficiency, residual sugar, and yeast population by the one-way analysis of variance (LSD, α = 0.05). The statistical software Statgraphics Centurion XVI.I was used for statistical procedures. Principal component analysis (PCA) was carried out using the Paleontological statistic Past 3.21 software.

## Results

### Yeast Identification

**Figure 2:**
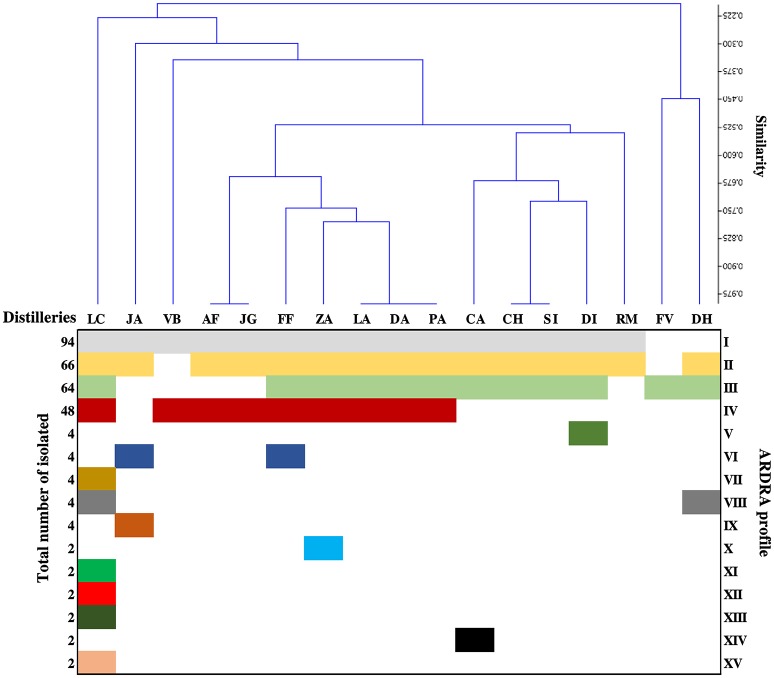
Clustering of distilleries based on the ARDRA profile detected in each sample. The letters RM, LC, PA, FV, DA, DI, LA, JA, SI, JG, DH, CH, CA, VB, AF, ZA, and FF correspond to the symbology assigned to each distillery. The numbers I to XV represent an ARDRA profile.

The yeasts population in each distillery was examined during the initial, middle, and final stage of the fermentation. A total of 304 yeast strains were isolated and subjected to molecular identification. After ARDRA analysis, 15 restriction patterns were detected. Figure 2 shows a clustering of the distilleries in terms of the restriction profile of the isolated strains. This Figure shows a clear predominance of profiles I, II, III, and IV. In general, distilleries were characterized by the presence of only 3 or 4 profiles. The only exception was LC distillery, which included 10 restriction patterns. It was also observed that some profiles were associated with a single distillery (Figure 2). The clustering analysis also showed that the ARDRA profile of distilleries LA, DA, and PA were grouped together, as well as AF and JG and CH and SI distilleries (Figure 2). Profiles (I to IV) containing the highest number of isolates, were directly identified by comparing sequences of the D1/D2 domain of 26S rRNA gene with those available in the GenBank (Table 1). The remaining groups (V to XV) including 2 or 4 isolates were considered as sporadic yeast species and they were not identified. The isolates were identified as (Table 1): *P. kudriavzevii* (group I), *P. manshurica* (group II), *S. cerevisiae* (group III), and *K. marxianus* (group IV). Among them, *P*. *kudriavzevii* was the most abundant species (94 isolates), followed by *P. manshurica* (66 isolates), *S*. *cerevisiae* (64 isolates), and *K. marxianus* (48 isolates), all the isolated showing the same D1/D2 ARDRA profile, corresponding to the same species.

**Table 1:** Grouping and identification of yeast isolates by ARDRA and sequencing of 26S rDNA D1/D2 domain.

**ARDRA group**	**Isolates^a^**	**AP^b^ (bp)**	**Restriction fragment length (bp)**			**Sequence length^c^ (bp)**	**Accession number^d^**	**Closest related type strains for GenBank/accession number**	**Identity^e^ %**
			***Hae* III**	***Hinf* I**	***Hha* I**			
I	94	640	245 + 215	400 + 130 + 110	230 + 165 + 155	545	KT875209	*P. kudriavzevii/* MG245854	100
II	66	635	245 + 215	395 + 130 + 110	215 + 150 + 140	509	KT875210	*P. manshurica*/ JF912077	100
III	64	615	325 + 160 + 130	220 + 185	615	460	KT875211	*S. cerevisiae* YMA 19Y/LT718652	100
IV	48	610	435 + 170	370 + 190	610	588	KT875208	*K. marxianus*/ MG245846	99

a *Number of strains with the same ARDRA pattern*.

b *AP is the 26S rDNA D1/D2 domain amplified product size in base pairs (bp)*.

c *Sequence length is the size of the D1/D2 domains of the 26S rDNA of the strains amplified with universal imers NL1 and NL4*.

d *Accesion number under which the nucleotide sequence was deposited in GenBank*.

e *Identity is the percentage of identical nucleotides in the sequence of the D1/D2 domains of 26S rDNA and the sequence with the best hit in the GenBank database. The ARDRA groups are Pichia kudriavzevii (group I), Pichia manshurica (group II), Saccharomyces cerevisiae (group III), Kluyveromyces marxianus (group IV)*.

### Yeast Species Distribution

According to Figure 1, predominant yeast populations did not show a specific preferential growth region. It was observed that the four yeast species were present in all regions except in San Juan del Rio, here *S. cerevisiae* and *K. marxianus* was not detected (Figure 1). Therefore, occurrence of predominant yeast between regions was not correlated with the geographic distance.

Table S1 in the supplementary material shows the presence and absence of the yeast species most commonly isolated from the distilleries. *Pichia kudriavzevii* and *P. manshurica* were the species most frequently encountered in the fermentation process. They were detected in 88% of distilleries, whereas, *S. cerevisiae* and *K. marxianus* were identified in 70 and 53% of distilleries respectively (Table S1 in supplementary material). Although there were many distilleries (FF, ZA, LC, PA, DA, and LA) with the same yeast species composition, their incidence in different stages of fermentation was dissimilar. For example, in the FF distillery, *P. kudriavzevii* and *P. manshurica* were isolated at the beginning of fermentation, while in the final stage, they were replaced by *S. cerevisiae* and *K. marxianus*. Although distilleries were located in the same municipality, their yeast distribution profile during fermentation (initial, middle, and final stages) differed. Results revealed the importance of *S. cerevisiae* and no-*Saccharomyces* yeasts in natural mezcal fermentation. Thus, no-*Saccharomyces* were exclusively isolated from the AF, JG, and JA distilleries. While in the DI and FV distilleries, only *S. cerevisiae* was detected, and yeast of this genus were also present in the CA, LC, PA, SI, and CH distilleries.

### Fermentative Capacity

**Figure 3:**
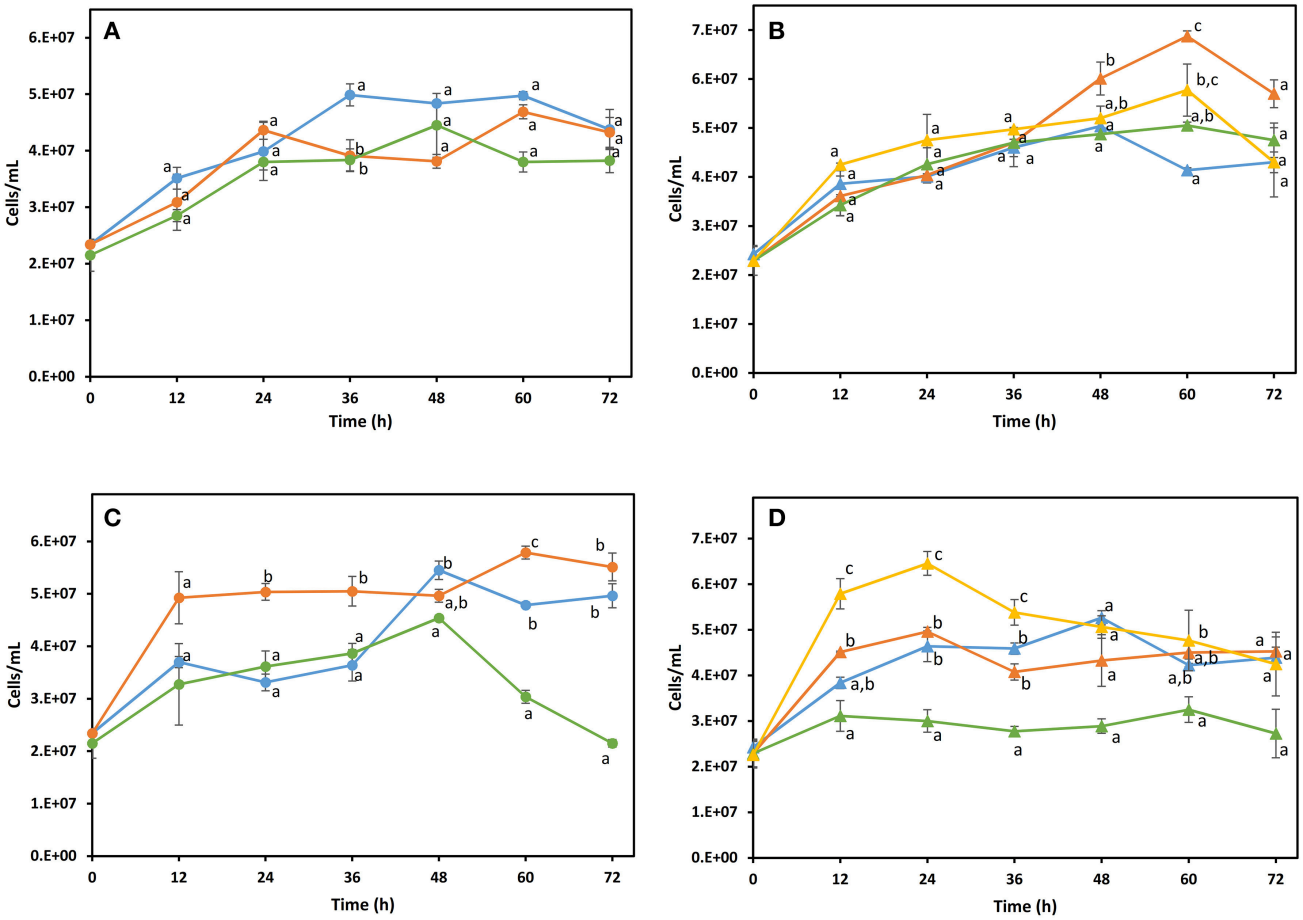
Growth kinetics of yeasts in filtered maguey juice **(A,B)** and maguey juice with bagasse **(C,D)**. Pure culture **(A,C)**: 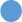 *K. marxianus* PA16, 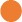 *P. kudriavzevii* JA10, 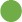 *S. cerevisiae* DI14. Mixed culture **(B,D)**: 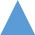 *S. cerevisiae* DI14 + *P. kudriavzevii* JA10,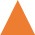 *K. marxianus* PA16 + *P. kudriavzevii* JA10,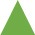 *S. cerevisiae* + *K. marxianus* PA16,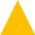 *S. cerevisiae* DI14 + *K. marxianus* PA16 + *P. kudriavzevii* JA10. Different letters on the points located at the same time show significant differences according to the analysis of variance at *p* ≤ 0.05 (LSD test).

Natural fermentation of maguey juice analyzed in this study, was characterized by the occurrence of the three predominant genera: *Pichia, Saccharomyces*, and *Kluyveromyces*, which suggests their importance in mezcal fermentation. Based on this information, the fermentative capacity, growth, and volatile compound production of these yeast species in maguey juice was investigated. *S*. *cerevisiae* and *K. marxianus* were selected because they are species commonly reported in maguey juice fermentation, producing both mezcal and tequila. While, *P*. *kudriavzevii* was selected as a representative of the *Pichia* genus, as this species has been studied for its high fermentative ability (Dhaliwal et al., 2011; Gallardo et al., 2011). JA10 (*P. kudriavzevii*), PA16 (*K. marxianus*), and DI14 (*S. cerevisiae*) strains were randomly taken for this analysis. The effects of pure and mixed cultures of these yeasts were evaluated in filtered maguey juice (MJ) and maguey juice with bagasse (MJB). In order to understand the production of the majority and normative compounds in mezcal, fermentation was initiated with the same biomass concentration in both pure and mixed cultures. In the mixed culture all yeast species were found in the same proportion. Figure 3 shows the kinetics of the growth of yeast. In the MJ medium, the yeast population in pure cultures did not show a significant statistical difference (Figure 3A). The highest cell concentration (7 × 10^7^ cell/mL) was reached by the non-*Saccharomyces* yeast grown in the MJ medium (Figure 3B). *P.kudriavzevii* showed higher growth in maguey juice than the growth reached by *S.cerevisiae* and *K. marxianus* (Figure 3C). Maguey juice with bagasse, the mixed culture containing all yeast species, reached the highest microbial population (Figure 3D). Pure cultures of *S. cerevisiae* DI14 and *K. marxianus* PA16 consumed 90% of the sugars, while *P. kudriavzevii* JA10 only consumed 38%. But, when this strain (JA10) was mixed with *S. cerevisiae* or *K. marxianus*, the sugar consumption was enhanced and 80% was consumed. Similar behavior was observed in both culture media (MJ and MJB). As a consequence of the low sugar consumption, *P. kudriavzevii* produced a low ethanol concentration (13 g/L). Pure culture of *K. marxianus* produced the highest concentration of ethanol (32 g/L) in MJ medium (Table 2). The ethanol production of pure cultures of *S. cerevisiae* (21.78 g/L) and *P. kudriavzevii* (11.63 g/L) in the MJB media, was improved when each of these strains were combined with *K. marxianus* (Table 2).

**Table 2:** Fermentative capacity of pure and mixed culture of *P. kudriavzevii* (JA10), *K. marxianus* (PA16), and *S. cerevisiae* (DI14) in MJ and MJB media.

**Strains**	**Culture media**	**Initial sugar**	**Residual**	**Final yeast population**	**Ethanol**	**Ethanol yield**	**Ethanol**	**Efficiency**
		**(g/L)**	**sugar (g/L)**	**(cells/mL)**	**Production**	**(Yp/s)^a^**	**productivity**	**(%)^c^**
					**(g/L)**		**(g/Lh)**^b^
*S.c*	MJ	73.36 ± 0.00	7.36 ± 3.14^abc^	3.83*E*+07 ± 2.12*E*+06^b^	24.85 ± 0.89^def^	0.38 ± 0.00^bcde^	0.34 ± 0.01^def^	73.72 ± 0.87^bcde^
*K.m*	MJ	76.32 ± 0.00	6.62 ± 0.00^ab^	4.38*E*+07 ± 3.54*E*+06^bc^	32.31 ± 0.50^g^	0.47 ± 0.01^fg^	0.46 ± 0.01^g^	90.71 ± 1.41^f^
*P.k*	MJ	76.32 ± 0.00	44.91 ± 6.68^g^	4.33*E*+07 ± 7.07*E*+05^bc^	13.93 ± 2.29^ab^	0.44 ± 0.02*e*^f^	0.19 ± 0.03^ab^	87.19 ± 4.29^ef^
*S.c-K.m*	MJ	79.29 ± 0.00	15.52 ± 0.00^d^	4.75*E*+07 ± 3.54*E*+06^cd^	20.99 ± 2.79^cd^	0.33 ± 0.0^abc^	0.29 ± 0.04^cd^	64.40 ± 8.56^abc^
*S.c-P.k*	MJ	73.36 ± 0.00	14.78 ± 1.05^d^	4.30*E*+07 ± 2.12*E*+06^bc^	18.15 ± 4.69^bc^	0.31 ± 0.07^ab^	0.25 ± 0.06^bc^	60.49 ± 14.57^ab^
*K.m-P.k*	MJ	79.29 ± 0.00	12.55 ± 0.00^cd^	5.70*E*+07 ± 2.83*E*+06^e^	26.63 ± 0.339^ef^	0.40 ± 0.00^cdef^	0.37 ± 0.01^ef^	78.08 ± 1.15^cdef^
*S.c-K.m-P.k*	MJ	79.29 ± 0.00	11.07 ± 0.00^bcd^	4.30*E*+07 ± 7.07*E*+06^bc^	27.38 ± 0.00^fg^	0.40 ± 0.00^cdef^	0.38 ± 0.00^fg^	78.53 ± 0.00^cdef^
*S.c*	MJB	73.36 ± 0.00	25.51 ± 0.55^e^	2.15*E*+07 ± 7.07*E*+05^a^	21.78 ± 2.23^cde^	0.45 ± 0.04^f^	0.30 ± 0.03^cde^	89.01 ± 8.10^f^
*K.m*	MJB	76.32 ± 0.00	2.91 ± 1.05^a^	4.96*E*+07 ± 2.30*E*+06^cde^	28.64 ± 0.67^fg^	0.39 ± 0.00^cdef^	0.40 ± 0.01^fg^	76.34 ± 0.69^cdef^
*P.k*	MJB	76.32 ± 0.00	35.54 ± 1.05^f^	5.51*E*+07 ± 2.65*E*+06^de^	11.63 ± 1.28^a^	0.28 ± 0.04^a^	0.16 ± 0.02^a^	55.94 ± 7.59^a^
*S.c-K.m*	MJB	79.29 ± 0.00	12.52 ± 0.00^cd^	2.73*E*+07 ± 5.30*E*+06^a^	28.32 ± 2.00^fg^	0.42 ± 0.03^def^	0.39 ± 0.03^fg^	83.05 ± 5.89^def^
*S.c-P.k*	MJB	73.36 ± 0.00	21.45 ± 6.29^e^	4.39*E*+07 ± 2.30*E*+06^bc^	20.75 ± 2.90^cd^	0.40 ± 0.01^cdef^	0.29 ± 0.04^cd^	78.14 ± 1.46^cdef^
*K.m-P.k*	MJB	79.29 ± 0.00	7.36 ± 1.05^abc^	4.53*E*+07 ± 3.18*E*+06^bc^	29.27 ± 3.79^fg^	0.41 ± 0.04^def^	0.41 ± 0.05^fg^	79.57 ± 9.16^def^
*S.c-K.m-P.k*	MJB	79.29 ± 0.00	5.13 ± 0.00^a^	4.30*E*+07 ± 7.07*E*+06^bc^	26.39 ± 3.51^ef^	0.36 ± 0.04^abcd^	0.37 ± 0.05^ef^	69.65 ± 9.27^abcd^

*These values are the mean ± standard deviation of two independent experiments. Different lowercase letters in the same column show significant differences according to the analysis of variance at p ≤ 0.05 (LSD test). MJ, maguey juice; MJB, maguey juice including bagasse; nd, non-detected. P. k, Pichia kudriavzevii; S. c, Saccharomyces cerevisiae; K. m, Kluyveromyces marxianus. Residual sugar, ethanol, and cell concentration were quantified at the end of fermentation (72 h)*.

a *Ethanol yield (Yp/s) was calculated as grams of ethanol produced per gram of utilized sugar*.

b *Ethanol productivity was calculated by ratio of ethanol production and fermentation time (72 h)*.

c *The efficiency of sugar conversion was calculated by ratio of the experimental yield (Yp/s) and the theoretical yield (0.511 g/g) multiplied by 100*.

*S. cerevisiae* presented a higher ethanol yield and productivity in MJB than the yield reached in the MJ medium. Conversely, non-*Saccharomyces* (*K. marxianus* and *P. kudriavzevii*) showed a higher yield and efficiency in the MJ medium. In addition, the presence of *S. cerevisiae* in the mixed culture of *K. marxianus* or *P. kudriavzevii* in the MJB medium, made the ethanol production more efficient (Table 2). *K. marxianus* proved to be a yeast species with great fermenting potential in maguey juice, with a high efficiency (90.71%) and an ethanol yield of about 0.47 g/g. In general, results revealed that maguey juice fermentation using pure cultures improved productivity. While, the fermentation of maguey juice with bagasse using a mixed culture, favored the productivity (Table 2). All fermentations were performed at 72 h. This time was established based on the consumption of sugar by *K. marxianus* as a reference. When the concentration of residual sugars reached a value < 5%, all fermentations were stopped, even if all sugars were not consumed.

### Volatile Compounds Production

The most important volatile compounds produced during mono and co-culture fermentations of maguey juice are presented in the Table 3. The highest concentration of higher alcohols was found to be about 108 mg/L (sum of the three higher alcohols reported in the Table 3) using the mixed cultures of *P. kudriavzevii* and *S. cerevisiae*. All pure and mixed cultures were characterized by isobutyl and isoamyl alcohol production. *P. kudriavzevii* produced twice the concentration of isobutyl alcohol (49 mg/L) than *S. cerevisiae* (27 mg/L) and *K. marxianus* (23 mg/L) produced. The production of these compounds was favored in maguey juice without bagasse (Table 3). Thus, the strain JA10 (*P. kudriavzevii*) produced the highest concentration of isobutyl alcohol (49 mg/L) in the MJ medium, however it strains produced only 26 mg/L in the MJB. Isobutyl alcohol concentration in fermentations using *S. cerevisiae* DI14 in the pure culture, mixed culture of *S. cerevisiae* with *P. kudriavzevii*, and the mixture of *K. marxianus* and *P. kudriavzevii* was also higher in the MJ media than those produced in the MJB media (Table 3). Similarly, *P. kudriavzevii* produced a higher concentration of ethyl acetate (283 mg/L) than *S. cerevisiae* or *K. marxianus*. *S. cerevisiae* DI14 produced concentrations of ethyl acetate < 5 mg/L, and *K. marxianus* did not produce it at all. Isobutyl alcohol and ethyl acetate concentration produced by *S. cerevisiae* and *K. marxianus* were improved when they were mixed with *P. kudriavzevii*. This strain also produced high ethyl acetate concentrations (280 mg/L), while *S. cerevisiae* produced concentrations lower than 5 mg/L, and in fermentations performed with *K. marxianus* this ester was not detected. This finding suggests that ester production depends on the yeast species. Our results also show that the ester type produced depend on the presence of bagasse in the maguey juice. Therefore, in maguey juice without bagasse, the ethyl acetate production was promoted. Conversely, ethyl lactate production was supported in maguey juice with bagasse (Table 3). In this study, under our fermentation conditions, methanol, butanol, sec-butanol, 1-propanol, and acetic acid was not identified, and acetaldehyde was detected only in some fermentations at concentrations below 5 mg/L.

**Table 3:** Concentration of volatile compounds produced by single and mixed cultures of *P. kudriavzevii* (JA10), *K. marxianus* (PA16), and *S. cerevisiae* (DI14) in MJ and MJB media.

	**Culture medium**	**Ethyl acetate**	**Ethyl lactate**	**Isobutyl alcohol**	**Isoamyl alcohol**	**Amyl alcohol**
		**mg/L**	**mg/L**	**mg/L**	**mg/L**	**mg/L**
S.c	MJ	< 5	nd	27.02 ± 0.73^c^	63.45 ± 5.65^g^	nd
K.m	MJ	nd	nd	23.23 ± 1.35^ab^	42.73 ± 6.45^bc^	nd
P.k	MJ	283.37 ± 4.76^f^	nd	49.19 ± 0.94^f^	39.38 ± 2.52^b^	5.29 ± 0.11^a^
S.c-K.m	MJ	nd	17.81 ± 2.0^3a^	23.67 ± 0.82^abc^	45.46 ± 0.64^c^	nd
S.c-P.k	MJ	42.027 ± 0.92^a^	nd	34.9 ± 0.40^d^	60.95 ± 0.67^fg^	nd
K.m-P.k	MJ	115.35 ± 2.33^e^	nd	39.62 ± 1.94^e^	51.54 ± 0.123^d^	5.48 ± 0.03^b^
S.c-K.m-P.k	MJ	103.17 ± 3.10^d^	nd	43.19 ± 2.81^e^	60.38 ± 0.88*e*^fg^	5.38 ± 0.12^ab^
S.c	MJB	< 5	20.81 ± 1.59^a^	23.21 ± 0.12^ab^	53.79 ± 1.63^d^	5.35 ± 0.22^ab^
K.m	MJB	nd	68.98 ± 9.86^b^	22.47 ± 0.22^a^	41.53 ± 0.44^bc^	nd
P.k	MJB	47.90 ± 1.27^a^	nd	26.17 ± 1.47^bc^	24.27 ± 0.19^a^	nd
S.c-K.m	MJB	nd	nd	26.99 ± 1.21^c^	54.34 ± 3.54^de^	nd
S.c-P.k	MJB	56.54 ± 10.13^b^	20.89 ± 1.25^a^	31.45 ± 1.26^d^	54.7 ± 0.12*d*^ef^	nd
K.m-P.k	MJB	75.37 ± 4.76^c^	20.69 ± 0.97^a^	34.54 ± 3.68^d^	54.47 ± 3.23^def^	nd
S.c-K.m-P.k	MJB	77.38 ± 3.36^c^	nd	31.78 ± 2.52^d^	52.50 ± 2.12^d^	5.37 ± 0.10^ab^

*These values are the mean ± standard deviation of two independent experiments. Different lowercase letters in the same column show significant differences according to the analysis of variance at p ≤ 0.05 (LSD test). MJ, maguey juice; MJB, maguey juice including bagasse; nd, non-detected. P. k, pichia kudriavzevii; S. c, saccharomyces cerevisiae; K. m, kluyveromyces marxianus. All compounds were quantified at the end of fermentation (72 h). The calibration range for all compounds were in a range of 5 to 200 mg/L*.

### PCA Analysis

**Figure 4:**
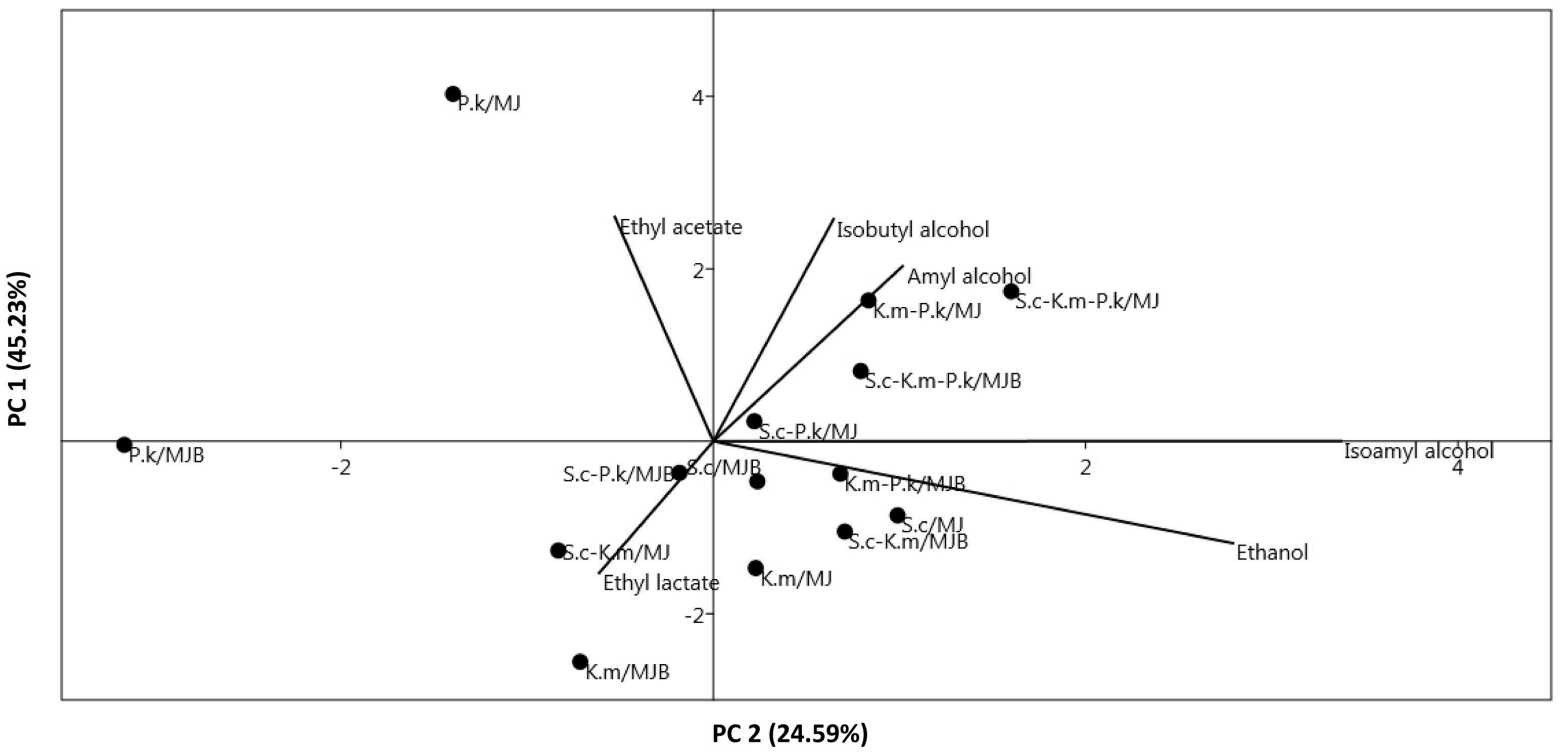
Principal components analysis (PCA) plot of volatile compounds produced and correlated with each pure and mixed inoculum (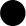; P. k, pichia kudriavzevii; S. c, saccharomyces cerevisiae; K. m, kluyveromyces marxianus) and culture medium (MJ, maguey juice; MJB, maguey juice including bagasse).

A PCA analysis was carried out to observe the relationship between the different variables. *P. kudriavzevii* was correlated with ethyl acetate in the MJ medium (Figure 4). Ethyl lactate production was correlated with the MJB medium. Higher alcohols had a correlation with fermentation of maguey juice without bagasse (MJ medium). Ethanol production was correlated with *S. cerevisiae* and *K. marxianus*.

## Discussion

### Yeast Identification and Distribution

The present study was performed in artisanal distilleries located in Oaxaca's regions. This State produces about 80% of mezcal within the Appellation of Origin (Mezcal Regulatory Council, 2018). Here, mezcals are mainly produced through artisanal methods, where fermentation occurs naturally. In this study, we investigated 9 regions and 17 distilleries (Figure 1) to build a broader understanding of predominant yeasts. The restriction patterns of D1/D2 region of 26S rDNA allowed species differentiation of common yeasts in the natural fermentation of mezcal. The use of this DNA region was already used by Baleiras-Couto et al. (2005) for differentiation of yeast species by monitoring the vinification process, which demonstrated its utility for clustering strains at a species level by a restriction analysis. *P. kudriavzevii, P. manshurica, S. cerevisiae*, and *K. marxianus* were the most frequently found species in the explored distilleries. With these isolates, we designed starters as pure or mixed cultures. The results showed a significant presence of non-*Saccharomyces* yeast in mezcal fermentations. This finding suggest that these species play an important role in the natural process. The predominant yeast population found in this study was different than those reported in other States, such as San Luis Potosí and Durango (Verdugo Valdez et al., 2011; Páez-Lerma et al., 2013). For example, *P. manshurica* and *P. kudriavzevii* appear to be two exclusive yeast species of Oaxaca State, while *S. cerevisiae* and *K. marxianus* are two common species in mezcal (Verdugo Valdez et al., 2011; Páez-Lerma et al., 2013), as well as in tequila fermentation (Lachance, 1995). Therefore, predominant yeast population in each distillery could be considered as a distinctive signature that gives a particular characteristic to the mezcal produced there. The predominance of non-*Saccharomyces* could be explained by the temperature inside the fermentation vats, which was around 40°C. This environmental condition supports the growth of thermotolerant non-*Saccharomyces* yeasts, such as *P. kudriavzevii* and *K. marxianus*. *P. kudriavzevii* has been reported as a thermotolerant yeast, with an optimal temperature of fermentation of 40 to 42°C (Dhaliwal et al., 2011; Gallardo et al., 2011). Moreover, *K. marxianus* has been characterized by its ability to tolerate temperatures about 40°C (Limtong et al., 2007; Hu et al., 2012). Comparable temperatures can be reached in the vats during maguey juice fermentation. Also, the predominance of *K. marxianus* in maguey juice fermentation could be explained by its capacity to grow on fructans, as a result of its fructanase activity (Arrizón et al., 2012). The prevalence of these yeasts depends on their ability to adapt to the environmental conditions of the fermentation vats, such as the sugar concentration (250 g/L), pH (4), ethanol (60 g/L), and temperature (40°C). Salvadó et al. (2011) showed that *S. cerevisiae* became the main species during the final stage of the fermentation process, when the ethanol concentration reached 9–10% v/v. Thus, in environments with ethanol concentrations below 9%, *S. cerevisiae* reduced its competitiveness and non-*Saccharomyces* may prevail (Salvadó et al., 2011). The above could explain the predominance of non-*Saccharomyces* species in mezcal fermentation, since ethanol concentration in this process is about 4–6% v/v.

The distribution of yeasts in the different distilleries revealed the importance of both non-*Saccharomyces* species and *S. cerevisiae* in the natural fermentation. In some instances only non-*Saccharomyces* were isolated, while in others they coexisted with *S. cerevisiae*. The dispersion of yeasts depends on several factors, such as the skin of the harvested magueys from different fields and the use of sweet cooked maguey, which promotes the indirect transmission of microorganisms through insect vectors (Lachance, 1995). During the cooked maguey's milling stage, human manipulation and uncontrolled conditions also allowed the spread and proliferation of microorganisms. These predominant yeasts have probably been established throughout the natural fermentation of maguey juice, and they have adhered to the walls of the wooden vats, equipment, and tools used in the process, and in each palenque's surroundings, similar to that reported in wineries (Garijo et al., 2008).

### Fermentative Capacity of Predominant Yeast

In general, results revealed that non-*Saccharomyces* yeast growth was slightly greater than that of *S. cerevisiae*, this helps us understand the predominance of these group of microorganisms in the natural fermentation of mezcal. These yeast species are probably better adapted to the maguey juice than *S. cerevisiae*, due to characteristics that are advantageous for its growth, such as the capacity to assimilate fructans; saponinasa activity, which prevent the microbial inhibition caused by saponins, compounds commonly present in the maguey plant (Alcázar et al., 2017); and rapid growth rate (Fonseca et al., 2008). It has been reported that a pure culture of *K. marxianus* grows and ferments the maguey juice as well as, or better than *S. cerevisiae* (López-Alvarez et al., 2012; Lopez et al., 2014; Segura-García et al., 2015). In accordance with previous studies we found that *K. marxianus* produced higher ethanol concentration, ethanol yield, productivity, and efficiency than that produced by the others yeast species assessed. Different *K. marxianus* strains isolated from maguey juice fermentation have demonstrated high fermentative capacity (López-Alvarez et al., 2012; Amaya-Delgado et al., 2013). López-Alvarez et al. (2012) suggested that *K. marxianus* UMPe-1 yeast isolated from mezcal fermentation can be suitable for maguey must fermentation at an industrial level. On the other hand, the sluggish fermentation performed by *P. kudriavzevii* could be attributed to the fermentation temperature (30°C) used in this trial, considering that this yeast has an optimal fermentation temperature of about 40 and 42°C (Dhaliwal et al., 2011; Gallardo et al., 2011).

Findings suggest that the presence of bagasse in maguey juice fermentation had no influence on ethanol production, it depends rather on the pure or mixed yeast strains used as inocula. However, it had a positive effect on the ethanol yield, which could be due to it acting as a support, where the yeast cells are immobilized, such as previously demonstrated in sugar cane bagasse (Santos et al., 2008), which promotes mass transfer and impacts the ethanol yield (Yu et al., 2010; Genisheva et al., 2011). In addition, maguey juice and the bagasse form a semisolid matrix, similar to a mud, which generates a more anaerobic environment than that in plain maguey juice, which can promote the conversion of sugar to ethanol in *S. cerevisiae*. On the contrary, the ethanol fermentation of *K. marxianus* is favored under aeration conditions and high inulin concentration (Gao et al., 2015). In general, results indicated that the fermentation of maguey juice with bagasse for artisanal mezcal production, could be favored by a mixed culture; and fermentation to produce a mezcal category, which is performed using only maguey juice, could be supported by a pure culture.

### Volatile Compounds Produced During Maguey Juice Fermentation

Higher alcohols, acetaldehyde, and methanol concentrations are regulated by the NOM-070 (NOM-070-SCFI-2016, 2017). Higher alcohols and esters are the most abundantly produced in maguey juice fermentation (Díaz-Montaño et al., 2008; Segura-García et al., 2015). It was reported that the production of these metabolites depends on the yeast species, as well as the coexistence with other species, the temperature and the carbon and nitrogen concentration (Molina et al., 2007; Saerens et al., 2010; Duarte et al., 2013; Liu et al., 2016). Under the studied conditions, methanol was not detected, probably due to its dependency on the pectin content of the maguey plant (Pinal et al., 2009), therefore, during the fermentation process it was not produced. Methanol is mainly produced during the maguey cooking, in this stage the methoxylated pectins are hydrolyzed and converted into this alcohol (Pinal et al., 2009). Aldehydes are also regulated by NOM-070, of which acetaldehyde is the most abundant. This compound contributes with aromas of green apple, fresh cut grass and nuts (Jackowetz et al., 2011). Acetaldehyde is a precursor of ethanol, so it is a major metabolic intermediary in yeast during fermentation (Swiegers et al., 2005). The low concentration of this compound in our fermentation could be attributed to the fact that determination was performed in the final stage of the fermentation (72 h). Evidence has shown that higher acetaldehyde concentrations are found in the adaptation stage and growth of the yeast, after which, the concentration decreases as a result of its reuse (Jackowetz et al., 2011).

In alcoholic beverages, higher alcohols can have both a positive and negative effect, depending on the concentration (Liu et al., 2016). The origin of these alcohols is associated with the metabolism of the amino acids. The production of isobutyl alcohol and isoamyl alcohol depends on the presence of valine and leucine in the culture medium, respectively (Dickinson et al., 1998; Swiegers et al., 2005). Both amino acids are abundant in some maguey species, such as *Agave tequilana* (Díaz-Montaño et al., 2008). This helps to explain the predominance of these alcohols in the fermentation. On the other hand, butanol, sec-butanol, and propanol were not detected in the fermentations and conditions studied, perhaps due to a lack of its precursor. Our findings suggest that the concentration of higher alcohols in maguey juice fermentation depends on the culture medium, the yeast species and the mixtures of yeast species. The esters production also depends on the yeast species. Some authors have demonstrated that non-*Saccharomyces* yeast produce higher amounts of esters than those produced by *S. cerevisiae* (Segura-García et al., 2015). In general, our results are in accordance with those reported by Amaya-Delgado et al. (2013), who found that volatile compounds produced in maguey juice fermentation, depend on the strain used as an inocula.

In this study, the importance of non-*Saccharomyces* yeasts and their contribution in the fermentation of mezcal was revealed. The data obtained in this study on the fermentative capacity and the ability to produce volatile compounds, helped us to understand the presence of the non-*Saccharomyces* yeast in fermentation where *S. cerevisiae* was not detected. Although *S. cerevisiae* did not predominate in some distilleries, non-*Saccharomyces* have the ability to produce ethanol with a yield greater or equal to those produced by *S. cerevisiae*. In addition, non-*Saccharomyces* contribute to the production of aroma and flavor compounds, such as esters and higher alcohols.

## Conclusions

Predominant yeast populations in the mezcal fermentation was mainly composed of *Pichia, Kluyveromyces*, and *Saccharomyces* genera. Ethanol production, yield, productivity, and efficiency of maguey juice fermentation results, showed that *K. marxianus* could be considered a suitable starter in artisanal mezcal production. Production and concentration of ethyl acetate, ethyl lactate, and amyl alcohol were dependent on the yeast species and the culture medium. While, production of other normative compounds, such as acetaldehyde, propanol, butanol, sec-butanol were not detected with the yeasts strain and the conditions evaluated. Single and mixed cultures revealed their influence on volatile compound production, suggesting that the yeasts combination could contribute to improving the organoleptic characteristics of artisanal mezcal.

## Author Contributions

FR-T, CW, and HN-C designed the project. HN-C collected the samples. HN-C performed the experiments. HN-C and JS-U contributed with the molecular identification of yeasts. HN-C generated and analyzed the data. HN-C wrote the paper. all authors reviewed the paper.

### Conflict of Interest Statement

The authors declare that the research was conducted in the absence of any commercial or financial relationships that could be construed as a potential conflict of interest.
