# Supplementary material for: Predominant Yeasts During Artisanal Mezcal Fermentation and Their Capacity to Ferment Maguey Juice
Source: Front Microbiol. 2018 Dec 6;9:2900. doi: 10.3389/fmicb.2018.02900 (PMC6291486; doi:10.3389/fmicb.2018.02900)
Supplement: Supplementary file 1 [file Table_1.DOCX]

Supplementary Material

Predominant yeasts during artisanal mezcal fermentation and their capacity to ferment maguey juice

Hipócrates Nolasco-Cancino, Jorge A. Santiago-Urbina, Carmen Wacher and Francisco Ruíz-Terán1*

*** Correspondence:** Corresponding Author: panchote@unam.mx

# Supplementary Table

**Table S1.** Distribution of predominant yeast species in the mezcal fermentation throughout different regions within the Appellation of Origin in Oaxaca State, Mexico

| District^a^ | Municipalities^b^ | Distillery | Stages of fermentation/isolated yeast species | | | | | | | | | | | | | | | |
| --- | --- | --- | --- | --- | --- | --- | --- | --- | --- | --- | --- | --- | --- | --- | --- | --- | --- | --- |
|  |  |  | Initial | | | |  | Middle | | | | | |  | Final | | | |
|  |  |  | *P. k* | *P. m* | *S. c* | *K. m* |  | *P. k* | | *P. m* | | *S. c* | *K. m* |  | *P. k* | *P. m* | *S. c* | *K. m* |
| Tehuantepec | Magdalena Tequisistlán | FF | ✓ | ✓ | - | - |  | ✓ | - | | - | | ✓ |  | - | - | ✓ | ✓ |
| Yautepec | Santa María Ecatepec | ZA | ✓ | ✓ | - | ✓ |  | - | ✓ | | ✓ | | ✓ |  | - | ✓ | - | - |
|  |  | AF | - | - | - | ✓ |  | - | ✓ | | - | | - |  | ✓ | ✓ | - | - |
|  | San Juan Lajarcía | CA | nd | nd | nd | nd |  | ✓ | ✓ | | ✓ | | - |  | ✓ | ✓ | ✓ | - |
|  |  | VB | nd | nd | nd | nd |  | ✓ | - | | - | | ✓ |  | ✓ | - | - | ✓ |
| Ocotlán | Santa Catarina Minas | LC | ✓ | ✓ | - | ✓ |  | ✓ | - | | ✓ | | - |  | - | ✓ | ✓ | - |
|  |  | RM | nd | nd | nd | nd |  | ✓ | ✓ | | - | | - |  | ✓ | ✓ | - | - |
|  | San Baltazar Chichicapam | FV | - | - | ✓ | - |  | - | - | | ✓ | | - |  | - | - | ✓ | - |
|  |  | PA | ✓ | - | - | ✓ |  | - | ✓ | | - | | ✓ |  | - | ✓ | ✓ | ✓ |
| Tlacolula | Santiago Matatlán | DA | ✓ | - | - | ✓ |  | ✓ | ✓ | | ✓ | | ✓ |  | ✓ | - | - | ✓ |
|  |  | LA | ✓ | - | ✓ | - |  | nd | nd | | nd | | nd |  | ✓ | ✓ | - | ✓ |
|  |  | DI | ✓ | ✓ | ✓ | - |  | ✓ | - | | ✓ | | - |  | - | - | ✓ | - |
|  | San Dionidisio Ocotepec | SI | ✓ | ✓ | ✓ | - |  | ✓ | ✓ | | ✓ | | - |  | ✓ | ✓ | ✓ | - |
|  |  | JG | ✓ | ✓ | - | ✓ |  | ✓ | ✓ | | - | | ✓ |  | ✓ | ✓ | - | ✓ |
|  | San Juan del Rio | JA | ✓ | ✓ | - | - |  | ✓ | - | | - | | - |  | ✓ | - | - | - |
|  | San Baltazar Guelavila | CH | ✓ | ✓ | ✓ | - |  | ✓ | ✓ | | ✓ | | - |  | ✓ | ✓ | ✓ | - |
|  |  | DH | - | - | ✓ | - |  | - | ✓ | | - | | - |  | - | ✓ | - | - |

^a^ District is the territorial division which includes several municipalities. ^b^ A municipality is the territory defined by fixed limits. *P. k*: *Pichia kudriavzevii*; *P. m*: *Pichia manshurica*; *S. c*: *Saccharomyces cerevisiae*; *K. m*: *Kluyveromyces marxianus*; ✓: presence; -: Absence; nd: not determined.
